# M. Trousseau’s Method of Tamponing

**Published:** 1854-09

**Authors:** 


*M. Trousseau's method of Tamponing.*—In a case of hemorrhage recently admitted at Hotel Dieu, the patient having aborted at two or three months, M. Trousseau resorted to the tampon after the following mode: The material used was carded cotton. This was not introduced as is ordinarily done, in portions completely separate, but a series of small tampons were attached to a string at equal distances, the whole presenting the appearance of the tail of a kite. Each of these tampons were dipped in cerate, and successively introduced, until the vagina was filled, and the flow of blood entirely arrested. After the lapse of ten hours the tampons were withdrawn and fresh ones introduced. A third and last introduction was made the following morning. *Charpie* is ordinarily used for tamponing in Paris. This substance, as remarked by M. Trousseau, is very hygrometic, and therefore poorly adapted to this purpose. Moreover, it becomes hard and wounds the parts with which it is in contact. Cotton, on the other hand, especially if dipped in a fatty substance, is impermeable to the blood; is soft, may be pressed into a mass with great facility, and makes compression without injury. The arrangement of a series of tampons on a string has the advantage of facilitating both the introduction and the extraction, particularly the latter.

After removing the tampons in this case, M. T. directed injections of *warm* water. He has been led to employ warm water, instead of cold, to arrest hemorrhages in consequence of some experiments made with M. Leblanc. According to these experiments, when blood is commingled with water at the temperature of 0, (Reamur,) it does not coagulate; but when commingled with water at 40° (R,) or higher, coagulation takes place. Clinical experience has verified, in his hands, the correctness of the inference from these facts, that injections of warm water, far from increasing hemorrhage as is commonly supposed, have a contrary effect.

---

*Salivation as a Diagnostic Sign distinguishing Variola from Varioloid in the early stage.*—M. Trousseau stated in a recent clinical lecture, that salivation is a constant symptom during the early period of the disease in variola, and the absence of this symptom in varioloid is invariable. Hence, according to him, the differential diagnosis may be made out by this symptom, before the disease has advanced sufficiently far to determine the variety of the disease by the symptoms. If this be reliable, it is the more valuable because the symptoms of the early stage in varoloid are often quite as violent as in grave cases of variola. To determine whether a patient is to pass through varioloid or variola is, of course, a point of not a little consequence with reference to the prognosis.

